# Physiological and metabolic responses in Kök‐Börü horses: Correlations with game outcomes

**DOI:** 10.1002/vms3.1457

**Published:** 2024-04-30

**Authors:** Ali Rişvanlı, İsmail Şen, Kanat Canuzakov, Askarbek Tülöbayev, Abuzer Taş, Ruslan Saklykov, Nezahat Ceylan, Ünal Türkçapar, Ulanbek Alimov, Arina Kazakbayeva, Ayday Cunuşova, Nur Abdımnap Uulu, Burak Fatih Yuksel, Mert Turanli, Muhammed Uz, Metin Bayraktar

**Affiliations:** ^1^ Faculty of Veterinary Medicine Kyrgyz‐Turkish Manas University Bishkek Kyrgyzstan; ^2^ Department of Obstetrics and Gynaecology Faculty of Veterinary Medicine Fırat University Elazig Türkiye; ^3^ Faculty of Sports Science Kyrgyz‐Turkish Manas University Bishkek Kyrgyzstan; ^4^ Department of Surgery Faculty of Veterinary Medicine Yuzuncu Yil University Van Türkiye; ^5^ Department of History Faculty of Humanities Kyrgyz‐Turkish Manas University Bishkek Kyrgyzstan; ^6^ Department of History Faculty of Humanities Ataturk University Erzurum Türkiye; ^7^ Faculty of Sports Science Kahramanmaras Sutcu Imam University Kahramanmaras Türkiye; ^8^ Faculty of Tourism Kyrgyz‐Turkish Manas University Bishkek Kyrgyzstan; ^9^ Department of Zootechny Faculty of Veterinary Medicine Fırat University Elazig Türkiye

**Keywords:** horse, Kök‐Börü, metabolism, physiology, stress

## Abstract

**Background:**

The aim of this study was to examine variations in stress, metabolic, and physiological parameters of horses used in the traditional equestrian team sport of Kök‐Börü in relation to winning and losing outcomes.

**Material and methods:**

To accomplish this, blood samples were taken from horses on four different teams who participated in two separate games, both before and after game. These samples were used to measure levels of cortisol, ACTH, beta‐endorphin, adrenaline, noradrenaline, triiodothyronine (T3), and thyroxine (T4) via species‐specific commercial ELISA kits. The autoanalyzer tested biochemical and hematological parameters. The gathered data were then analyzed statistically based on the teams' winning or losing status.

**Results:**

The results suggested that winning teams had lower MID, red blood cell, HGB, RDW‐SD, HCT, platelet distribution width, and creatine kinase values post‐game in comparison to their pre‐game state. Conversely, mean corpuscular hemoglobin concentration (MCHC), mean corpuscular hemoglobin (MCH), and CREA values increased in the winning teams' post‐game. Additionally, horses in the winning teams showed a decrease in cortisol, beta‐endorphin, and ACTH levels post‐game but increased levels of adrenaline and T3. Considering the pre‐game values, it was found that GRA and Cl levels were lower in the winning teams. Before the game, adrenaline and T3 levels were higher in the winning teams. No significant difference was observed in post‐game hematological parameters between the teams. However, post‐game K, adrenaline, and noradrenaline levels were higher among the winning teams' horses, while cortisol and beta‐endorphin levels were heightened in horses from the losing side.

**Conclusion:**

In conclusion, significant differences were not observed in the distribution of hematological and biochemical parameters of horses following the Kök‐Börü games, regardless of the outcome. However, decreased post‐game cortisol, ACTH, and beta‐endorphin levels in winning teams may suggest better stress management abilities among these horses.

## Graphical abstract

‐ Kök‐Börü, a favoured traditional equestrian sport in Asia countries.

‐ Stress, metabolic and physiological parameters of horses used in Kök‐Börü.

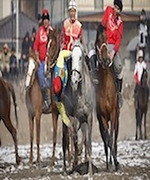

## 1 INTRODUCTION

Equestrian games and sports are an integral part of societal culture worldwide, especially in Central Asia. Notable among these are equestrian javelin (Cirit), Polo and Kök‐Börü. Kök‐börü, in particular, is still quite popular in countries like Turkmenistan, Kyrgyzstan and Kazakhstan, where it is structured into super leagues, first leagues and lower leagues. The game takes place on expansive flat fields, with two teams that can range from 5 to 15 players each (Gül et al., 2018; Yücel, 2010). The rules may differ slightly between countries, but the main objective remains the same – to score points by hurling previously dried skins of lamb or goat into the opposing team's goal. The team that scores the most points within a set period wins the game (Kurt et al., 2017; Kafkasyalı, 2005).

Two primary physiological axes contribute to stress in mammals: the sympathetic–adrenal–medulla axis and the hypothalamic–pituitary–adrenal axis. The former is linked to immediate stress responses, prompting the activation of our sympathetic nervous system. This results in an elevated heart rate, higher blood pressure, the release of catecholamines (adrenaline and noradrenaline), and a slowed‐down gastrointestinal process. The activation of this axis leads to an instantaneous reaction to stress (Brown, 1994).

**FIGURE 1:**
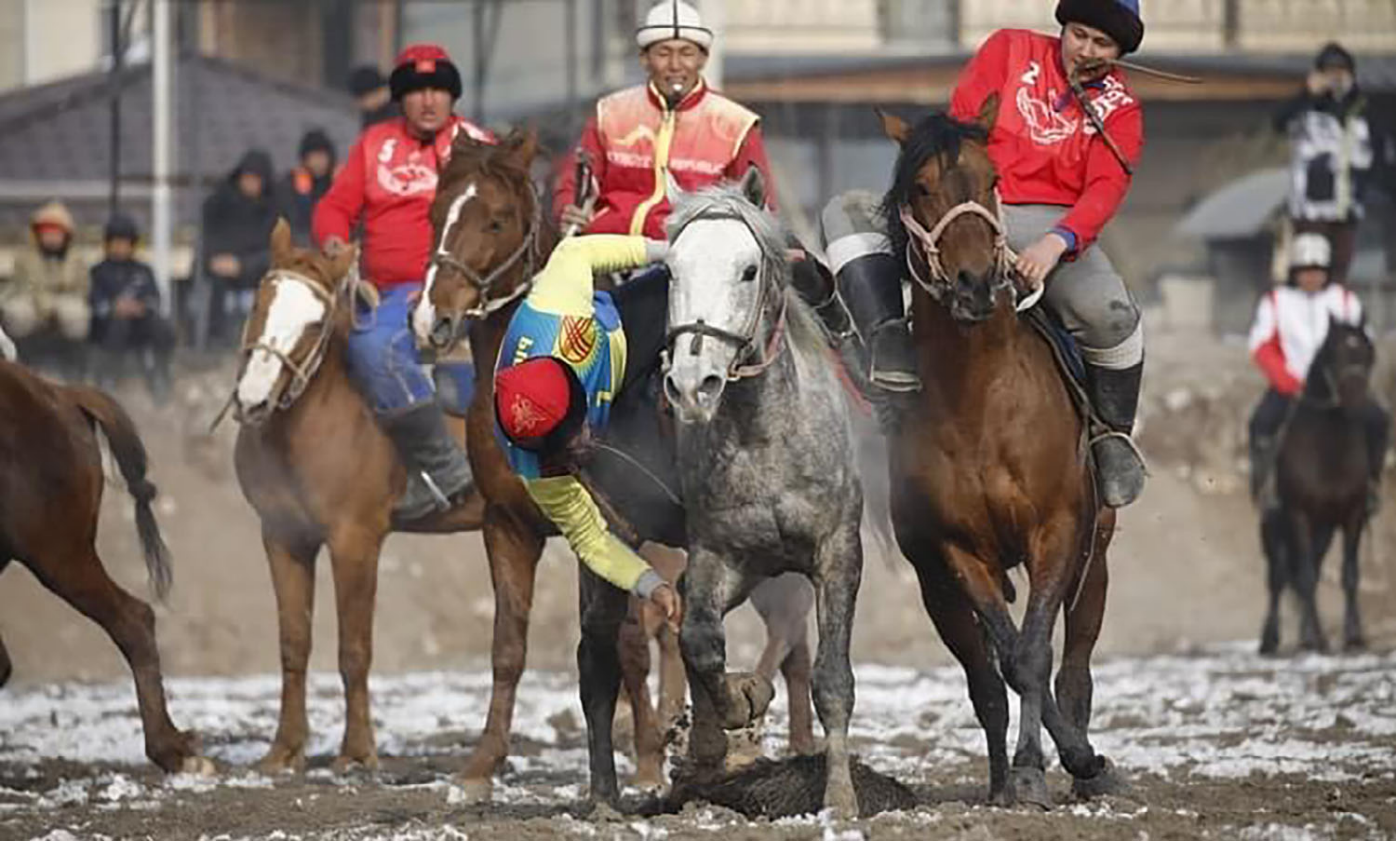
Kök‐Börü game.

On the contrary, the hypothalamic–pituitary–adrenal axis provokes long‐lasting effects by prompting the release of corticosteroids from the adrenal cortex. Figure 1 This induces the release of adrenocorticotropic hormone (ACTH) from the pituitary gland through the secretion of corticotropin‐releasing hormone. Given that most cells express cortisol receptors, cortisol can affect a variety of organs and systems in the body, including metabolic, cardiovascular and immune responses (Dhabhar, 2014; McEwen, 1998).

Concentrations of catecholamines and peripheral cortisol are often used to evaluate the body's adaptive responses to stress. As they are the initial endocrine response to stress, these parameters have been widely studied in humans and animals (Möstl & Palme, 2002; Manteca, 1998; Hada et al., 2003; Kyrou & Tsigos, 2007).

Catecholamines, also known as ‘stress hormones’, are responsible for various metabolic changes during both rest and activity (Zouhal et al., 2008). In stressful situations, the sympathetic nervous system activates to facilitate rapid responses across a variety of functions, including respiration, endocrine and cardiovascular responses (Cuniberti et al., 2012).

The endogenous opioids group comprises many chemicals, including the widely studied enkephalins, beta‐endorphins and dynorphins. These opioids play a significant dual role in the body: They help regulate food intake and modulate sexual and social activities. Just like in humans, they hold analgesic qualities in horses and assist in managing pain thresholds (Cayado et al., 2006). Evolved as part of the organism's defence mechanism against stress, the pain‐relief function of opioid receptors is also regarded to have immunomodulatory effects. This is especially observable in the context of the immune system's regulation (Golynski et al., 2011).

The goal of this study is to examine how winning or losing in the traditional equestrian game Kök‐Börü affects various stress, metabolic and physiological factors in horses.

## 2 MATERIALS AND METHODS

In this study, horses from four teams in the Kyrgyztan Kök‐Börü Super League, which competed at Kyrgyzstan‐Turkey Manas University's Ethnosport Facilities, were used as research subjects (Figure 1).

Our project included 40 clinically healthy male Kök‐Börü horses from the Kyrgyz breed. Each horse was about 3–4 years old and weighed between 500 and 600 kg. They were all raised, fed, and trained under the same conditions.

### 2.1 Blood sampling procedure

Blood samples were taken from ten horses on each team, both half an hour before the game and within 30 min of its conclusion. Samples were drawn from the horses’ jugular vein into 10 mL tubes for serum separation. Concurrently, blood was also collected into 5 mL EDTA tubes from these horses for haematological analysis. Overall, blood was drawn from 40 horses in total.

The serum samples were preserved at −80°C in a deep freezer until tested. The EDTA blood samples were analysed within 2 h of collection.

Blood samples were taken from horses pre and post‐game to analyse cortisol, ACTH, beta‐endorphin, adrenaline, noradrenaline, triiodothyronine (T3) and thyroxine (T4) levels.

The haematological tests conducted included measurements of parameters, such as white blood cell, red blood cell (RBC), haemoglobin (HGB), haematocrit (HCT), mean corpuscular volume, mean corpuscular haemoglobin (MCH), mean corpuscular haemoglobin concentration (MCHC), mean platelet volume, platelet distribution width (PDW), plateletcrit, red cell distribution width (RDW), platelet large cell ratio, mid‐range cells (MID), lymphocytes, granulocytes (GRA) and platelet count.

Biochemical parameters for serum were measured, including sodium (Na), potassium (K), chloride (Cl), creatinine (CREA), urea, total protein, albumin, magnesium (Mg), alkaline phosphatase (AP), creatine kinase (CK), alanine aminotransferase (ALT) and aspartate aminotransferase (AST).

### 2.2 Hormone analysis

We used commercial ELISA kits specific to each species to measure hormone levels in the collected blood serum. We measured cortisol (catalogue no: CEA462Ge, USCN Life Science Inc.), ACTH (catalogue no: CSB‐E18039Hs, CUBIO Innovation Center), beta‐endorphin (catalogue no: LS‐F25041, LSBio), adrenaline (catalogue no: CEA858Ge, USCN Life Science Inc.), noradrenaline (catalogue no: CEA907Ge, USCN Life Science Inc.), T3 (catalogue no: CEA453Ge, USCN Life Science Inc.) and T4 (catalogue no: CEA452Ge, USCN Life Science Inc.). We followed the ELISA test application steps outlined in the manufacturer's guide. After completing the process, we determined serum hormone levels by reading the plates at 450 nm with an ELISA reader (Bio‐Tek Instruments) (Can‐Şahna & Rişvanlı, 2015).

### 2.3 Biochemical analysis

Biochemical parameters, aside from electrolytes, were analysed using an automated chemistry analyser. An electrolyte analysis device determined the electrolyte levels (Safak et al., 2022).

### 2.4 Haematological analysis

Measurements for CBC and WCDC were taken using an automatic laser haematology analyser. Blood tests were conducted at the Kyrgyzstan‐Turkey Manas University University Central Laboratory, at the Veterinary Faculty Laboratories, or in private laboratories via service procurement (Safak et al., 2022).

Subsequently, the data obtained from horses pre and post‐game were statistically analysed based on winning and losing situations.

### 2.5 Statistical analyses

After the study concluded, all data on animal parameters was moved into the SPSS for Windows 26 software (IBM). The data's normal distribution was verified both statistically and graphically. Since it did not demonstrate a normal distribution or fulfil parametric test assumptions, nonparametric tests were used the Mann–Whitney *U* test for independent groups and the Wilcoxon test for dependent groups.

## 3 RESULTS

The haematological findings for the horses involved in the study are presented in Table 1. A brief explanation of Table 1 results follows:

**TABLE 1:** Haematological parameters for horses.

	Winning (1st and 4th teams)			Losing (2nd and 3rd teams)				
	Pre‐game	Post‐game	*p*	Pre‐game	Post‐game	*p*	*p* Pre‐game	*p* Post‐game
**WBC 10^9/L**	8.04 ± 0.48	8.92 ± 0.56	0.113	9.86 ± 0.76	10.11 ± 0.83	0.828	0.113	0.347
**LYM 10^9/L**	1.97 ± 0.13	2.13 ± 0.22	0.705	2.02 ± 0.14	1.98 ± 0.17	0.820	0.986	0.904
**MID 10^9/L**	0.44 ± 0.03	0.63 ± 0.07	**0.025**	0.57 ± 0.05	0.70 ± 0.07	0.099	0.056	0.408
**GRA 10^9/L**	5.62 ± 0.41	6.017 ± 0.40	0.068	7.61 ± 0.78	7.29 ± 0.86	0.691	**0.018**	0.469
**LYM %**	25.04 ± 1.49	23.41 ± 1.49	0.130	21.05 ± 1.61	21.59 ± 2.12	0.649	0.095	0.449
**MID %**	5.52 ± 0.25	6.96 ± 0.47	**0.012**	5.68 ± 0.34	7.21 ± 0.64	0.053	0.986	0.796
**GRA %**	69.44 ± 1.50	69.63 ± 1.79	0.740	73.27 ± 1.78	71.27 ± 2.66	0.609	0.138	0.593
**RBC 10^12/L**	9.65 ± 0.59	11.21 ± 0.60	**0.022**	10.79 ± 0.72	11.61 ± 0.42	0.267	0.359	0.210
**HGB g/L**	164.65 ± 8.97	186.65 ± 9.06	**0.031**	180.05 ± 11.56	192.94 ± 6.81	0.240	0.466	0.330
**MCHC g/L**	311.18 ± 0.93	307.53 ± 1.06	**0.031**	312.84 ± 1.49	306.77 ±0.79	**0.020**	0.691	0.295
**MCH pg**	16.97 ± 0.23	16.75 ± 0.27	**0.036**	16.74 ± 0.17	16.63 ± 0.14	0.068	0.428	0.655
**MCV fl**	54.48 ± 0.65	54.45 ± 0.75	0.065	53.55 ± 0.52	54.23 ± 0.42	0.068	0.437	0.754
**RDW‐CV %**	15.85 ± 1.34	16.18 ± 0.15	0.055	15.96 ± 0.15	16.10 ± 0.11	0.337	0.656	0.643
**RDW‐SD Fl**	33.58 ± 0.48	34.75 ± 0.63	**0.023**	33.16 ± 0.41	34.54 ± 0.26	**0.002**	0.616	0.869
**HCT %**	52.22 ± 2.92	60.70 ± 2.94	**0.031**	56.94 ± 3.40	62.84 ± 2.16	0.112	0.537	0.308
**PLT 10^9/L**	155.76 ± 8.41	153.82 ± 7.16	0.755	139.32 ± 7.55	142.22 ± 6.85	0.679	0.093	0.262
**MPV fl**	6.93 ± 0.10	6.99 ± 0.07	0.076	7.04 ± 0.11	6.93 ± 0.15	1.000	0.533	0.610
**PDW %**	14.43 ± 0.54	15.66 ± 0.46	**0.025**	15.48 ± 0.39	14.85 ± 0.84	0.109	0.215	0.256
**PCT %**	0.12 ± 0.01	0.11 ± 0.01	1.00	0.11 ± 0.002	0.11 ± 0.01	1.000	0.148	0.524
**P‐LCR %**	7.19 ± 0.49	7.28 ± 0.39	0.312	7.03 ± 0.39	6.45 ± 0.34	0.593	0.772	0.229

Abbreviations: GRA, granulocytes; HCT, haematocrit; HGB, haemoglobin; LYM, lymphocytes; MCH, mean corpuscular haemoglobin; MCHC, mean corpuscular haemoglobin concentration; MID, mid‐range cells; PCT, plateletcrit; PDW, platelet distribution width; P‐LCR, platelet large cell ratio; PLT, platelet count; RBC, red blood cell; RDW, red cell distribution width.

Bold value indicates statistical significance.

In winning teams, the MID value was found to be lower post‐ game (6.96% ± 0.47%) compared to pre‐ game (5.52% ± 0.25%) (*p* = 0.012). The RBC value was also lower in winning teams post‐game ((11.21 ± 0.60)×10^12^/L) compared to pre‐game ((9.65 ± 0.59)×10^12^/L) (*p* = 0.022). Similarly, the HGB value was lower in winning teams post‐game (186.65 ± 9.06 g/L) compared to pre‐game (164.65 ± 8.97 g/L) (*p* = 0.031). However, the MCHC value was higher in winning teams post‐game (307.53 ± 1.06 g/L) compared to pre‐game (311.18 ± 0.93 g/L) (*p* = 0.031). The MCH value was also higher in winning teams post‐game (16.75 ± 0.27 pg) compared to pre‐game (16.97 ± 0.23 pg) (*p* = 0.036). The RDW‐SD value was lower in winning teams post‐game (34.75 ± 0.63 Fl) compared to pre‐game (33.58 ± 0.48 Fl) (*p* = 0.023). The HCT value was lower in winning teams post‐game (60.70% ± 2.94%) compared to pre‐game (52.22% ± 2.92%) (*p* = 0.031). Additionally, the PDW value was lower in winning teams post‐ game (15.66% ± 0.46%) compared to p game (14.43% ± 0.54%) (*p* = 0.025).

In losing teams, it was found that the MCHC value was higher pre‐game (312.84 ± 1.49 g/L) compared to post‐ game (306.77 ± 0.79 g/L) (*p* = 0.020). Moreover, in losing teams, the RDW‐SD value pre‐game (33.16 ± 0.41 Fl) was lower than pgame (34.54 ± 0.26 Fl) (*p* = 0.002).

When comparing pre‐game GRA values, it was observed that winning teams (5.62 ± 0.41 10^9^/L) had lower values compared to losing teams (7.61 ± 0.78 10^9^/L).

However, there was no statistically significant difference in post‐game haematological parameters between winning and losing teams’ horses.

The study's biochemical data for horses is summarized in Table 2. Here is a brief explanation of the results:

**TABLE 2:** Biochemical parameters in horses.

	Winning (1st and 4th teams)			Losing (2nd and 3rd teams)				
	Pre‐game	Post‐game	*p*	Pre‐game	Post‐game	*p*	*p* Pre‐game	*p* post‐game
**CK**	321.82 ± 15.62	441.13 ± 21.69	**0.001**	357.53 ± 31.38	411.38 ± 35.88	**0.023**	0.639	0.585
**ALBUMIN**	31.70 ± 0.87	31.61 ± 0.70	0.501	29.73 ± 0.59	30.48 ± 0.84	0.327	0.081	0.351
**TP**	98.39 ± 32.82	69.00 ± 2.48	0.121	67.58 ± 1.57	69.49 ± 1.32	0.231	0.516	0.691
**ALT**	18.79 ± 1.20	21.37 ± 1.48	0.163	17.88 ± 2.34	21.05 ± 2.59	**0.004**	0.161	0.407
**AST**	441.30 ± 25.06	442.37 ± 28.85	0.460	455.07 ± 79.92	466.54 ± 77.46	**0.000**	0.124	0.426
**AP**	248.51 ± 69.62	193.26 ± 14.21	0.179	198.45 ± 13.24	212.17 ± 13.41	**0.005**	0.188	0.241
**CREA**	158.24 ± 65.88	118.00 ± 4.82	**0.016**	88.00 ± 2.63	115.06 ± 3.55	**0.000**	0.076	0.592
**UREA**	5.74 ± 0.13	5.68 ± 0.38	0.378	5.89 ± 0.27	6.33 ± 0.27	**0.002**	0.824	0.397
**Na**	140.22 ± 0.56	139.71 ± 1.09	0.776	139.49 ± 0.36	137.87 ± 0.85	**0.019**	0.281	0.152
**K**	3.62 ± 0.10	3.65 ± 0.09	0.842	3.55 ± 0.10	3.56 ± 0.10	**0.046**	0.715	**0.031**
**Mg**	0.74 ± 0.03	0.64 ± 0.02	0.002	0.66 ± 0.02	0.60 ± 0.02	**0.001**	0.049	0.152
**Cl**	91.04 ± 0.61	91.35 ± 1.02	0.605	93.99 ± 0.98	91.39 ± 1.16	0.117	**0.022**	0.796

Abbreviations: ALT, alanine aminotransferase; AP, alkaline phosphatase; AST, aspartate aminotransferase; CK, creatine kinase; CREA, creatinine; Na, including sodium.

Bold value indicates statistical significance.

For the winning teams, it was observed that the post‐game serum CK (441.13 ± 21.69, *p = 0.001) level was higher, whereas CREA (118.00 ± 4.82, *p = 0.016) was lower.

The horses from the losing teams showed higher post‐game serum levels of CK (411.38 ± 35.88, *p = 0.023), ALT (21.05 ± 2.59, *p = 0.004), AST (466.54 ± 77.46, *p = 0.000), AP (212.17 ± 13.41, *p = 0.005), CREA (115.06 ± 3.55, *p = 0.016), UREA (6.33 ± 0.27, *p = 0.002) and K (3.56 ± 0.10, *p = 0.046). However, post‐game serum levels of Na (137.87 ± 0.85, *p = 0.019) and Mg (0.60 ± 0.02, *p = 0.001) were lower.

When comparing the pre‐game Cl levels, it was observed that they were lower in the winning teams (91.04 ± 0.61, *p = 0.022) compared to the losing teams.

Comparing post‐game *K* levels, it was determined that they were higher in the winning teams (3.65 ± 0.09, *p = 0.031).

The study's hormonal data for horses is summarized in Table 3. An explanation of the results follows:

**TABLE 3:** Hormonal data for horses.

	Winning (1st and 4th teams)			Losing (2nd and 3rd teams)				
	Pre‐game	Post‐game	*p*	Pre‐game	Post‐game	*p*	*p* Pre‐game	*p* Post‐game
**Cortizol ng/mL**	109.09 ± 12.13	81.05 ± 6.11	**0.011**	93.17 ± 3.24	97.09 ± 1.89	0.246	0.843	**0.003**
**Beta‐endorfin pg/mL**	93.13 ± 17.51	36.80 ± 5.12	**0.005**	57.83 ± 4.30	91.29 ± 14.63	**0.049**	0.291	**0.000**
**Adrenaline pg/mL**	32.79 ± 3.74	941.21 ± 189.27	**0.003**	47.80 ± 1.22	34.08 ± 3.84	**0.002**	**0.000**	**0.004**
**Noradrenaline pg/mL**	271.43 ± 107.37	1202.11 ± 837.27	0.287	133.30 ± 23.86	108.93 ± 12.60	0.795	0.156	**0.019**
**ACTH pg/mL**	1509.17 ± 156.94	778.70 ± 244.38	**0.006**	1775.65 ± 83.14	2012.95 ± 84.36	0.113	0.210	0.001
**T3 pg/mL**	98.74 ± 16.32	621.13 ± 121.55	**0.001**	175.77 ± 20.93	112.06 ± 14.35	0.055	**0.008**	0.029
**T4 ng/mL**	157.08 ± 26.10	127.47 ± 27.87	0.586	222.87 ± 9.58	162.29 ± 16.67	**0.002**	0.069	0.907

Bold value indicates statistical significance.

In winning teams, post‐game serum cortisol (81.05 ± 6.11 ng/mL, *p* = 0.011), beta‐endorphin (36.80 ± 5.1 pg/mL, *p* = 0.005) and ACTH (778.70 ± 244.38 pg/mL, *p* = 0.006) levels were found to be lower compared to pre‐game levels. However, post‐game serum adrenaline (941.21 ± 189.27 pg/mL, *p* = 0.003) and T3 (621.13 ± 121.55 pg/mL, *p* = 0.001) levels were higher than pre‐game levels in winning teams.

For losing teams, post‐game serum beta‐endorphin levels (91.29 ± 14.63 pg/mL, *p* = 0.049) were higher compared to pre‐game levels. However, post‐game serum adrenaline (34.08 ± 3.84 pg/mL, *p* = 0.002) and T4 (162.29 ± 16.67 pg/mL, *p* = 0.002) levels were lower than pre‐game levels in winning teams.

Winning teams were observed to have higher levels of pre‐game adrenaline (941.21 ± 189.27 pg/mL, *p* = 0.000) and T3 (621.13 ± 121.55 pg/mL, *p* = 0.001) compared to losing teams.

The post‐game serum cortisol (97.09 ± 1.89 ng/mL, *p* = 0.003) and beta‐endorphin (91.29 ± 14.63 pg/mL, *p* = 0.000) levels were found to be higher in losing teams than in their winning counterparts. Likewise, post‐game serum adrenaline (941.21 ± 189.27 pg/mL, *p* = 0.004) and noradrenaline (1202.11 ± 837.27 pg/mL, *p* = 0.019) levels were more elevated in winning teams than in the teams that lost.

## 4 DISCUSSION

Kök‐Börü is a traditional equestrian team sport that has largely been examined only from historical and sociological angles. There's a notable literature void surrounding the link between the results of equestrian team sports and physiological parameters. Therefore, this study offers pioneering insights into this topic.

Exercise triggers numerous physiological and anatomical adaptations in horses, which are crucial for counteracting physiological stress induced by activity (Hinchcliff & Geor, 2004). Blood components, like muscle tissue, also undergo changes due to exercise. Biochemical shifts in the blood show alterations in the performance of various bodily systems (Balogh et al., 2001).

In a study by Ayvazoğlu et al. (2023) on Arabian racehorses, biochemical data were taken before and after a 30‐min training session. They reported pre‐training levels of creatine phosphokinase (CK‐MB), lactate dehyrogenase (LDH), aspartat aminotransferase (AST) and Alanin Amimotransferase (ALT) as 231.15 ± 8.96, 692.45 ± 34.12, 309.92 ± 18.48 and 11.83 ± 0.92 U/L, respectively. After training, the levels were 289.80 ± 10.96, 704.25 ± 22.03, 328.47 ± 19.58 and 15.24 ± 1.03 U/L, respectively. Studies on Thoroughbred racehorses reported increased LDH, AST and ALT after exercise (Allaam et al., 2014). Horses with piroplasmosis have shown similar increases in LDH and AST due to exercise (Bravo‐Barriga et al., 2022). In endurance horses, a significant hike in AST and ALT levels was found after a race compared to pre‐race measurements (Larsson et al., 2013). The rise in LDH post‐exercise is thought to be a result of oxidative peroxidation and is believed to be due to hypoxia‐induced damage to musculoskeletal cells and hepatocytes, depending on the intensity of the exercise (Jović et al., 2013). Our study found that in equestrian team sports, the post‐game serum CK level was higher, and the CREA level was lower in the horses from winning teams. In contrast, horses from losing teams had higher post‐game serum CK, ALT, AST, AP, CREA, UREA and K levels but lower Na and Mg levels. Pre‐game Cl levels of winning team horses were lower than those of losing teams. However, the post‐game *K* values were higher for winning teams. Therefore, no significant changes were observed in pre‐game and post‐game biochemical parameters based on victory or defeat in the traditional equestrian sport of Kök‐Börü.

In the study by Allaam et al. (2014) blood samples were taken from racehorses to measure changes in their haematological parameters (RBCs, PCV, Hb, total and differential leucocytic count) at 5, 10 and 60 min after running 1600 m. They found that these parameters increased at the 5‐min mark but returned to normal by the 60‐min mark. Despite extensive research, there is no data on how the haematological parameters of horses in team equestrian sports relate to performance outcomes. The findings from the current study reveal differences in certain parameters between winning and losing teams pre and post‐games. In winning teams, the levels of pre‐game MID (5.52% ± 0.25%), RBC ((9.65% ± 0.59)×10^12^/L), HGB (164.65 ± 8.97 g/L), RDW‐SD (33.58 ± 0.48 Fl), HCT (52.22% ± 2.92%) and PDW (14.43% ± 0.54%) were lower compared to their post‐game values. Conversely, pre‐game MCHC (311.18 ± 0.93 g/L) and MCH (16.97 ± 0.23 pg) values were higher. In contrast, losing teams showed a higher pre‐game MCHC value (312.84 ± 1.49 g/L) compared to the post‐game value (306.77 ± 0.79 g/L). The pre‐game RDW‐SD value (33.16 ± 0.41 Fl) was also lower than the post‐game value (34.54 ± 0.26 Fl). Furthermore, pre‐game GRA values were lower in winning teams (5.62 ± 0.41 10^9^/L) compared with losing teams (7.61 ± 0.78 10^9^/L). These observations suggest that variations in haematological parameters pre and post‐games in the traditional equestrian sport of Kök‐Börü do not significantly correlate with team performance.

Studies have examined the response of adrenaline and noradrenaline in horses to various diseases, surgical operations and physical exercises (Cuniberti et al., 2012; Ayala et al., 2012; Baragli et al., 2011). Cortisol is a commonly used indicator of acute stress in veterinary research, often used to assess short‐term stress from transportation or caregiving procedures. Healthy horses exhibit variable cortisol levels depending on the time of day, the season and their overall body condition, but their age and sex do not appear to affect these levels (Aurich et al., 2015; Hart et al., 2016; Cordero et al., 2012).

Cortisol levels peak within 10–20 min of stress occurrence following a circadian rhythm (Lay et al., 1992). During stressful events, horses particularly release beta‐endorphin into the bloodstream (Fazio et al., 2013). The release of opioids into the blood reportedly happens within 1 h of encountering stress. Beta‐endorphin is thought to manage the hypothalamic–pituitary–adrenal axis activity, thus regulating ACTH secretion. In horses, the secretion of beta‐endorphin aligns with the onset of a stress response and is believed to counteract cortisol's detrimental effects on the body (Golynski et al., 2011). No hormone data on horses’ performance in equestrian team sports concerning winning and losing outcomes was found despite numerous studies. However, in this study, we observed significant hormonal changes in winning teams. Following the game, cortisol (81.05 ± 6.11 ng/mL, *p* = 0.011), beta‐endorphin (36.80 ± 5.1 pg/mL, *p* = 0.005) and ACTH (778.70 ± 244.38 pg/mL, *p* = 0.006) levels dropped, whereas adrenaline (941.21 ± 189.27 pg/mL, *p* = 0.003) and T3 (621.13 ± 121.55 pg/mL, *p* = 0.001) levels rose compared to pre‐game measurements. A comparison of pre‐game adrenaline (941.21 ± 189.27 pg/mL, *p* = 0.000) and T3 (621.13 ± 121.55 pg/mL, *p* = 0.001) levels showed higher values in horses from winning teams than losses. Conversely, post‐game cortisol (97.09 ± 1.89 ng/mL, *p* = 0.003) and beta‐endorphin (91.29 ± 14.63 pg/mL, *p* = 0.000) concentrations were higher in losing teams. Similarly, post‐game adrenaline (941.21 ± 189.27 pg/mL, *p* = 0.004) and noradrenaline (1202.11 ± 837.27 pg/mL, *p* = 0.019) levels were higher in winning teams compared to their losses.

In conclusion, our data suggests that winning or losing in Kök‐Börü games does not significantly affect horses’ haematological or biochemical parameters. However, horses from winning teams displayed lower post‐game levels of cortisol, ACTH and beta‐endorphin, suggesting better stress management. Further extensive studies would reinforce this interpretation.

## AUTHOR CONTRIBUTIONS

*Conceptualization; Data curation; Formal analysis; Investigation; Methodology; Project administration; Resources; Software; Validation; Visualization; Writing—original draft; Writing—review and editing*: Ali Rişvanlı, İsmail Şen and Kanat Canuzakov. *Conceptualization; Funding acquisition; Investigation; methodology; Supervision; Writing—review and editing*: Askarbek Tülöbayev, Abuzer Taş, Nezahat Ceylan, Ünal Türkçapar and Ulanbek Alimov. *Investigation; methodology; Supervision*: Arina Kazakbayeva, Ayday Cunuşova and Nur Abdımnap Uulu. *Investigation; Methodology; Resources*: Burak Fatih Yuksel, Mert Turanli, Muhammed Uz and Metin Bayraktar.

## CONFLICT OF INTEREST STATEMENT

The authors declare no conflicts of interest related to this article.

### ETHICS STATEMENT

This project was approved by the Animal Ethics Committee at Kyrgyzstan‐Turkey Manas University (Document Date and Number: 20.01.2023‐2023/01).

### PEER REVIEW

The peer review history for this article is available at https://publons.com/publon/10.1002/vms3.1457.

## Data Availability

The data that support the findings of this study are available from the corresponding author upon reasonable request.

## References

[vms31457-bib-0021] Allaam, M. , Elseady, Y. , Nayel, M. , Elsify, A. , Salama, A. , Hassan, H. , Hassan, M. , & Kamar, A. (2014). Physiological and hemato‐chemical evaluation of thoroughbred race horse after exercise. International Journal of Advanced Veterinary and Medical Science, 8, 81–93.

[vms31457-bib-0027] Aurich, J. , Wulf, M. , Ille, N. , Erber, R. , von Lewinski, M. , Palme, R. , & Aurich, C. (2015). Effects of season, age, sex, and housing on salivary cortisol cntrations in horses. Domestic Animal Endocrinology, 52, 11–16. 25700267 10.1016/j.domaniend.2015.01.003

[vms31457-bib-0025] Ayala, I. , Martos, N. F. , Silvan, G. , Gutierrez‐Panizo, C. , Clavel, J. G. , & Illera, J. C. (2012). Cortisol, adrenocorticotropic hormone, serotonin, adrenaline and noradrenaline serum cntrations in relation to disease and stress in the horse. Research in Veterinary Science, 93, 103. 21641009 10.1016/j.rvsc.2011.05.013

[vms31457-bib-0020] Ayvazoğlu, C. , Kızıltpe, Ş. , Yaşar, Ü. , Yaşar, Z. G. , Demir, P. , & Tunc, A. C. (2023). Changes in cardiac troponin I (cTnI), T (cTnT), and some biochemical parameters in Arabian racehorses after training. South African Journal of Animal Science, 53, 1–6.

[vms31457-bib-0019] Balogh, N. , Gaal, T. , Ribiczeynè, P. S. , & Petri, A. (2001). Biochemical and antioxidant changes in plasma and erythrocytes of pentathlon horses before and after exercise. Veterinary Clinical Pathology, 30, 214–218. 12024305 10.1111/j.1939-165x.2001.tb00434.x

[vms31457-bib-0026] Baragli, P. , Sgorbini, M. , Casini, L. , Ducci, M. , & Sighieri, C. (2011). Early evidence of the anticipatory response of plasma catecholamine in equine exercise. Journal of Equine Veterinary Science, 31, 85–88.

[vms31457-bib-0022] Bravo‐Barriga, D. , Serrano‐Aguilera, F. J. , Barrasa‐Rita, R. , Habela, M. Á. , Chacón, R. B. , Ezquerra, L. J. , & Martín‐Cuervo, M. (2022). Effects of competitive ELISA‐positive results of piroplasmosis on the performance of endurance horses. Animals (Basel), 12, 637. 35268210 10.3390/ani12050637 PMC8909285

[vms31457-bib-0005] Brown, R. E. (1994). An introduction to neuroendocrinology. Cambridge University Press.

[vms31457-bib-0016] Can‐Şahna, K. , & Rişvanlı, A. (2015). Th1/Th2 cytokine balance and SOCS3 levels of female offspring born from rats with gestational diabetes mellitus. Kafkas Üniversitesi Veteriner Fakültesi Dergisi, 21, 837–840.

[vms31457-bib-0014] Cayado, P. , Muňoz‐Escassi, B. , Dominguez, C. , Manley, W. , Olabarri, B. , Sánchez de la Muela, M. , Castejon, F. , Marañon, G. , & Vara, E. (2006). Hormone response to training and competition in athletic horses. Equine Veterinary Journal, 36, 274–278. 10.1111/j.2042-3306.2006.tb05552.x 17402431

[vms31457-bib-0029] Cordero, M. , Brorsen, B. W. , & McFarlane, D. (2012). Circadian and circannual rhythms of cortisol, ACTH, and melanocyte‐stimulating hormone in healthy horses. Domestic Animal Endocrinology, 43, 317–324. 22717182 10.1016/j.domaniend.2012.05.005

[vms31457-bib-0013] Cuniberti, B. , Badino, P. , Odore, R. , Girardi, C. , & Re, G. (2012). Effects induced by exercise on lymphocyte—Adrenergic receptors and plasma catecholamine levels in performance horses. Research in Veterinary Science, 92, 116–120. 21168179 10.1016/j.rvsc.2010.11.002

[vms31457-bib-0007] Dhabhar, F. S. (2014). Effects of stress on immune function: The good, the bad, and the beautiful. Immunologic Research, 58, 193–210. 24798553 10.1007/s12026-014-8517-0

[vms31457-bib-0031] Fazio, E. , Medica, P. , Cravana, C. , & Ferlazzo, A. (2013). Hypothalamic‐pituitary‐adrenal axis responses of horses to therapeutic riding program: Effects of different riders. Physiology & Behavior, 118, 138–143. 23684906 10.1016/j.physbeh.2013.05.009

[vms31457-bib-0015] Golynski, M. , Krumrych, W. , & Lutnicki, K. (2011). The role of beta‐endorphin in horses: A review. Veterinary Medicine, 56, 423–429.

[vms31457-bib-0001] Gül, M. , Uzun, R. N. , & Çebi, M. (2018). A superficial glance at the traditional games and sports in the Turkic cultures. Turkish Studies Social Sciences, 13, 655–671.

[vms31457-bib-0010] Hada, T. , Onaka, T. , Takahashi, T. , Hiraga, A. , & Yagi, K. (2003). Effects of novelty stress on neuroendocrine activities and running performance in thoroughbred horses. Journal of Neuroendocrinology, 15, 638–648. 12787048 10.1046/j.1365-2826.2003.01042.x

[vms31457-bib-0028] Hart, K. A. , Wochele, D. M. , Norton, N. A. , McFarlane, D. , Wooldridge, A. A. , & Frank, N. (2016). Effect of age, season, body condition, and endocrine status on serum free cortisol fraction and insulin cntration in horses. Journal of Veterinary Internal Medicine, 30, 653–663. 26860336 10.1111/jvim.13839 PMC4913614

[vms31457-bib-0018] Hinchcliff, K. W. , & Geor, R. J. (2004). Integrative physiology of exercise. In K. W. Hinchcliff , Kaneps A. J. , R. J. Geor (Eds.), Equine sports medicine and surgery (pp 3–8). Saunders.

[vms31457-bib-0024] Jović, S. , Stevanović, J. , Borozan, S. , Dimitrijević, B. , & Milosavljević, P. (2013). Influence of physical activity of racehorses on lactate dehydrogenase and creatine kinase activities, and protein synthesis. Acta Veterinaria, 63, 549–568.

[vms31457-bib-0004] Kafkasyalı, A. (2005). Overview of Newroz tradition in the Turkish World. Journal of Graduate School of Social Sciences, 6, 149–172.

[vms31457-bib-0003] Kurt, T. , Kılıç, M. , Kılıç, M. N. , Özbayraktar, F. , Yücel, E. , & Kıvanç, C. (2017). Turkish sports history. State Books Publications.

[vms31457-bib-0011] Kyrou, I. , & Tsigos, C. (2007). Stress mechanisms and metabolic complications. Hormone and Metabolic Research, 39, 430–438. 17578760 10.1055/s-2007-981462

[vms31457-bib-0023] Larsson, J. , Pilborg, P. H. , Johansen, M. , Christophersen, M. T. , Holte, A. , Roepstorff, L. , Olsen, L. H. , & Harrison, A. P. (2013). Physiological parameters of endurance horses pre‐compared to post‐race, correlated with performance: A two‐race study from Scandinavia. International Scholarly Research Notices, 2013, 684353. 10.1155/2013/684353 PMC3791564 24167733

[vms31457-bib-0030] Lay, D. C. , Friend, T. H. , Bowers, C. L. , Grissom, K. K. , & Jenkins, O. C. (1992). A comparative physiological and behavioral study of freeze and hot‐iron branding using dairy cows. Journal of Animal Science, 70, 1121–1125. 1582942 10.2527/1992.7041121x

[vms31457-bib-0009] Manteca, X. (1998). Neurophysiology and assessment of welfare. Meat Science, 49, 205–218. 22060712

[vms31457-bib-0006] McEwen, B. S. (1998). Stress, adaptation, and disease: Allostasis and allostatic load. Annals of the New York Academy of Sciences, 840(1), 33–44. 9629234 10.1111/j.1749-6632.1998.tb09546.x

[vms31457-bib-0008] Möstl, E. , & Palme, R. (2002). Hormones as indicators of stress. Domestic Animal Endocrinology, 23, 67–74. 12142227 10.1016/s0739-7240(02)00146-7

[vms31457-bib-0017] Safak, T. , Yilmaz, O. , Risvanli, A. , & Akdeniz‐Incili, C. (2022). Hematological, serum biochemical results, and treatment approach of an Arabian mare with squamous cell carcinoma of the vulva‐case report. Arquivo Brasileiro de Medicina Veterinária e Zootecnia, 74, 525–529.

[vms31457-bib-0002] Yücel, M. U. (2010). Horse races of Kazakh Turks in the national games. Acta Turcica, 2, 353–375.

[vms31457-bib-0012] Zouhal, H. , Jacob, C. , Delamarche, P. , & Gratas‐Delamarche, A. (2008). Catecholamines and the effects of exercise, training and gender. Sports Medicine (Auckland, N.Z.), 38, 401–423. 18416594 10.2165/00007256-200838050-00004

